# Primary renal myxoid liposarcoma with pancreatic invasion on ^18^F-FDG PET/CT: first case report and literature review

**DOI:** 10.3389/fmed.2023.1235843

**Published:** 2023-07-27

**Authors:** Wenpeng Huang, Fangfang Chao, Yongbai Zhang, Liming Li, Yuan Gao, Yongkang Qiu, Jianbo Gao, Lei Kang

**Affiliations:** ^1^Department of Nuclear Medicine, Peking University First Hospital, Beijing, China; ^2^Department of Nuclear Medicine, The First Affiliated Hospital of Zhengzhou University, Zhengzhou, China; ^3^Department of Radiology, The First Affiliated Hospital of Zhengzhou University, Zhengzhou, China

**Keywords:** myxoid liposarcoma, kidney, pancreas, recurrence, computed tomography, ^18^F-FDG

## Abstract

**Background:**

Myxoid liposarcoma (MLS) is a rare malignant soft tissue sarcoma that predominantly manifests in the deep soft tissues of the extremities, particularly within the musculature of the thigh. Unlike other types of liposarcoma, MLS demonstrates a propensity for metastasis to atypical sites, including the lung parenchyma, soft tissues, retroperitoneum, mediastinum, breast, liver, thymus, lymph nodes, and bones. The definitive diagnosis primarily relies on histology with HE staining. Imaging modalities such as ultrasound, CT, MRI, and ^18^F-FDG PET/CT scans serve as valuable tools for tumor identification.

**Case report:**

A 57-year-old man presented with symptoms of abdominal distention and vomiting 1 month ago. Contrast-enhancement CT revealed a heterogeneous hypodense mass in the upper-middle part of the left kidney, displaying irregular morphology and protrusion towards the exterior of the kidney, with abundant blood supply and had a maximum dimension of approximately 10.7 cm × 9.0 cm. Additionally, a rounded soft tissue density was identified in the pancreatic body. Multiplanar reconstruction demonstrated a connection between the pancreatic lesion and the kidney mass. ^18^F-FDG PET/CT was conducted for staging, revealing significant growth of the lesion in the upper-middle part of the left kidney, extending beyond the kidney and infiltrating the pancreatic body. The lesion demonstrated remarkably high ^18^F-FDG uptake (SUVmax = 10.2, MTV = 136.13 cm^3^, TLG = 484.62). The postoperative pathological examination confirmed the diagnosis of MLS. On the 10th day post-surgery, the patient presented with tumor recurrence and underwent another surgical resection. Unfortunately, during the operation, the patient experienced a sudden cardiac arrest and died.

**Conclusion:**

Renal MLS with invasion into the pancreas is very rare in clinical practice. Due to the limited research on the utilization of ^18^F-FDG PET/CT in this particular context, given the rarity and low incidence of MLS, its role remains largely unexplored. As PET/CT imaging becomes increasingly prevalent, thorough imaging of disease sites becomes indispensable for the development of treatment protocols and the monitoring of treatment response.

## Introduction

Malignant tumors of adipocytic origin encompass well-differentiated liposarcoma, myxoid liposarcoma (MLS), dedifferentiated liposarcoma, pleomorphic liposarcoma, and myxoid pleomorphic liposarcoma (1). MLS is a rare malignant soft tissue sarcoma (STS) that accounts for approximately 30% of all liposarcomas and 10% of all STS (2, 3). The incidence of MLS reaches its peak during the fourth and fifth decades of life, affecting both genders equally (4). MLS predominantly manifests in the deep soft tissues of the extremities, particularly within the musculature of the thigh, and exhibits slow growth (5, 6). MLS is a sarcoma associated with a chromosomal translocation *t(12:16) (q13:p11)*, leading to the formation of the FUS-CHOP oncoprotein. This oncoprotein interacts with DNA promoters, causing dysregulation of downstream protein expression (7, 8). Unlike other types of liposarcoma, MLS exhibits a propensity for metastasis to atypical sites, encompassing the lung parenchyma, soft tissues (abdominal or chest wall), retroperitoneum, mediastinum, epidural space, breast, liver, thymus, pancreas, lymph nodes, and bones (9–14). Choosing the appropriate imaging modality is crucial for the accurate diagnosis and effective management of patients with STS (15). Positron emission tomography (PET) is a functional imaging technique that enables the assessment of tumor or physiologic tissue metabolism *in vivo* using positron-emitting radionuclides. Specifically, the uptake of fluorine-18 deoxyglucose (^18^F-FDG) is utilized to identify the heightened metabolic activity of tumorous cells through glucose metabolism (16). ^18^F-FDG PET/CT plays a significant role in the staging, restaging, detection of local recurrence and metastatic disease, as well as the prediction of prognosis and assessment of therapeutic response in STS (17, 18).

Here, we present a unique case of MLS of the left kidney invading the pancreas, exhibited a dismal clinical course and prognosis. In addition, we summarized the ^18^F-FDG PET/CT findings of MLS from the literature in Table 1 (19–29).

**Table 1:** Previous cases of myxoid liposarcoma with ^18^F-FDG PET/CT.

Case	Authors	Patient sex	Age	Clinical presentation	Primary sites	Max diameter (cm)	Grade	SUVmax	Invasion and metastasis	Management	Prognosis
1	Paladino et al. (19)	F	31	Acute back pain	The left thigh	NA	NA	NA	Vertebrae	Surgery + chemotherapy + radiotherapy + cellular therapy	Alive at 1 y
2	Lunn et al. (20)	F	37 y	NA	Right gluteus medius muscle	NA	II	3.9	NA	NA	NA
3	Lunn et al. (20)	M	48 y	NA	Proximal left thigh, subcutaneous	NA	II	2.2	NA	NA	NA
4	Lunn et al. (20)	M	37 y	NA	Right sartorius muscle	NA	I	2.8	NA	NA	NA
5	Lunn et al. (20)	M	15 y	NA	Proximal left thigh, subcutaneous	NA	I	1.6	NA	NA	NA
6	Lunn et al. (20)	F	42 y	NA	NA	NA	II	4.2	NA	NA	NA
7	Suzuki et al. (21)	F	48 y	NA	Intramuscular	3	NA	0.79	NA	NA	NA
8	Suzuki et al. (21)	M	36 y	NA	Subcutaneous	6	NA	1.29	NA	NA	NA
9	Suzuki et al. (21)	F	39 y	NA	Intermuscular	15	NA	1.9	NA	NA	NA
10	Suzuki et al. (21)	M	36 y	NA	Subcutaneous	9	NA	2.4	NA	NA	NA
11	Suzuki et al. (21)	M	42 y	NA	Intermuscular	22	NA	2.4	NA	NA	NA
12	Suzuki et al. (21)	M	42 y	NA	Intermuscular	16	NA	2.4	NA	NA	NA
13	Suzuki et al. (21)	F	79 y	NA	Intermuscular	25	NA	2.5	NA	NA	NA
14	Suzuki et al. (21)	F	59 y	NA	Intramuscular	17	NA	2.57	NA	NA	NA
15	Suzuki et al. (21)	F	78 y	NA	Subcutaneous	8	NA	3.1	NA	NA	NA
16	Sakamoto et al. (22)	M	43 y	NA	Left leg	NA	NA	NA	Right neck region, the retroperitoneum, lung and vertebrae	Surgery + chemotherapy	Alive at 10 y
17	Baffour et al. (23)	M	37 y	NA	Right sartorius muscle	NA	I	2.8	NA	NA	NA
18	Liu et al. (24)	M	29 y	A small nodule above the umbilicus	The left lobe of the liver	11.0	NA	3.1	Abdomen and pelvic cavities	Chemotherapy	Died after few weeks
19	Kudo et al. (25)	M	52 y	A slowly enlarging, painless mass at the dorsal aspect of the left foot	The deep portion of the plantar aspect of the left foot	8	NA	5.5	Para-aortic lymph node	Surgery + chemotherapy + radiotherapy	NA
20	Ramamurthy et al. (26)	F	53	Shortness of breath, orthopnea, a dry cough, and a low-grade intermittent fever	Right atrium	5.8	NA	3.4	Multiple lymph nodes and abdomen areas	NA	NA
21	Schwab et al. (27)	F	65	Low back pain	Popliteal	NA	III	Non-elevated FDG uptake in metastasis	The second lumbar vertebrae	Surgery + radiotherapy	Died
22	Shivdasani et al. (28)	F	56	A large painless swelling in posterior aspect of the left thigh	Left thigh	11	NA	5	None	Surgery + radiotherapy	Alive
23	Ozguven et al. (29)	M	48	NA	Left thigh	NA	NA	4.1	Pelvis, paravertebra, right gluteal region, and mesenteric region of abdomen	Surgery + chemotherapy	Alive at 3 y

NA, not available.

## Case presentation

A 57-year-old man presented with symptoms of abdominal distention and vomiting 1 month ago and has lost 15 kg since the onset of his illness. Physical examination revealed no abnormalities. Laboratory tests showed elevated levels of D-dimer (0.42 mg/L), fibrinogen (4.95 g/L), and C-reactive protein (141.50 mg/L), while tumor markers were within the normal range. The patient had a history of hypertension for 10 years and paroxysmal supraventricular tachycardia for 1 year.

**Figure 1:**
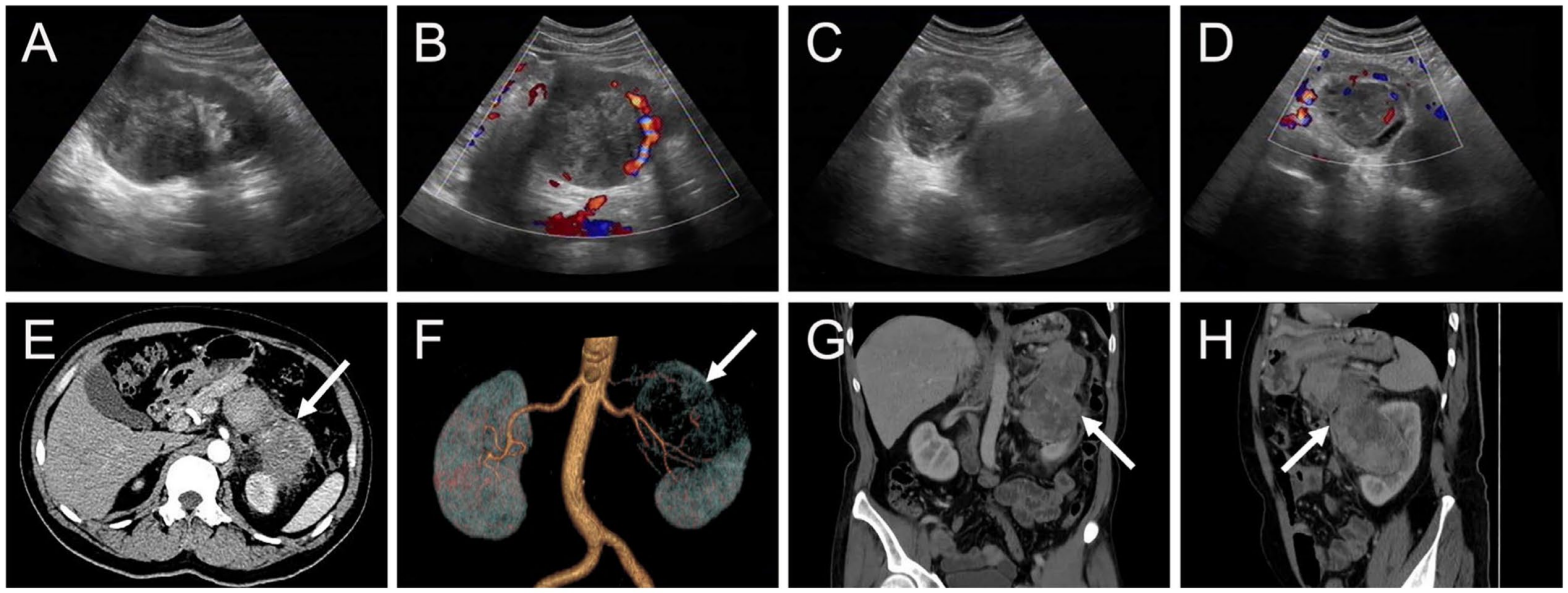
Ultrasound and contrast-enhanced computed tomography (CT) images of myxoid liposarcoma (MLS) of the left kidney invading the pancreas. **(A,B)** A heterogeneous hypoechoic mass measuring approximately 7.8 cm × 7.0 cm was observed in the upper middle part of the left kidney with indistinct borders. Color Doppler flow imaging (CDFI) revealed the presence of grade II flow signal within the mass. **(C,D)** Additionally, a cystic solid mass measuring approximately 4.8 cm × 4.4 cm was identified in the posterior part of the pancreas, immediately adjacent to the superior pole of the left kidney. CDFI indicated the presence of grade I blood flow signal within the mass. **(E)** The arterial phase transverse CT image depicted a protruding mass in the upper middle part of the left kidney, exhibiting extrarenal growth (long arrows), blurring of the perirenal fat gap, and significant enhancement (76 HU). **(F)** A volume rending (VR) image demonstrated abundant blood supply to the mass, with multiple branches of the left renal artery entering the lesion. **(G,H)** Coronal and sagittal images obtained from the venous phase displayed invasion of the pancreas by the left kidney mass (98 HU).

**Figure 2:**
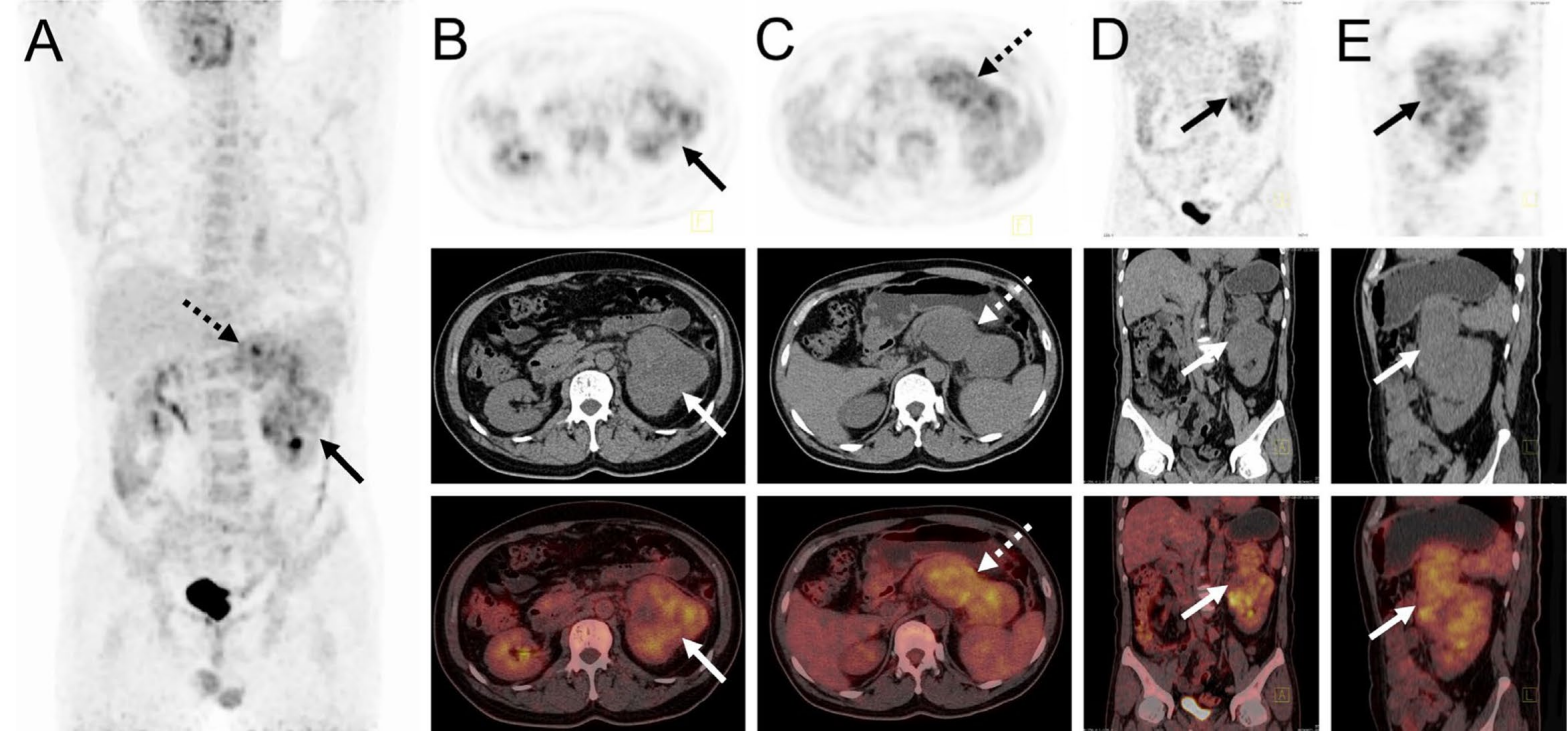
^18^F-FDG PET/CT images of myxoid liposarcoma of the left kidney invading the pancreas. **(A)** The anteroposterior 3-dimensional maximum intensity projection image (MIP) revealed increased metabolic activity in the left kidney (long arrows) and the pancreatic body region (dashed arrows). **(B)** Transverse images exhibited irregular morphology of the left kidney, accompanied by significantly high FDG uptake (SUVmax = 9.3). **(C)** Transverse images showed a pancreatic body lesion with significantly high FDG uptake (SUVmax = 10.2). **(D,E)** Coronal and sagittal images demonstrated an extra-renal protrusion of the left renal lesion and invasion of the pancreas.

The patient underwent an abdominal ultrasound examination, which revealed a heterogeneous hypoechoic mass in the upper middle part of the left kidney, measuring approximately 7.8 cm × 7.0 cm with unclear boundaries. Color Doppler flow imaging demonstrated grade II blood flow signal within the mass (Figures 1A,B). Additionally, a cystic solid mass measuring approximately 4.8 cm × 4.4 cm was detected in the posterior part of the pancreas, located immediately adjacent to the superior pole of the left kidney (Figures 1C,D). CT examination revealed a heterogeneous hypodense mass in the upper middle part of the left kidney, exhibiting irregular morphology and protrusion towards the exterior of the kidney, along with blurring of the perirenal fat gap. The lesion measured approximately 7.0 cm × 9.0 cm × 10.7 cm, showing significant enhancement (Figure 1E). The mass displayed rich blood supply, with visible branches of the left renal artery entering the mass (Figure 1F). Additionally, a rounded soft tissue density was identified in the pancreatic body. Multiplanar reconstruction revealed a connection between the pancreatic lesion and the mass in the left kidney (Figures 1G,H). ^18^F-FDG PET/CT was conducted for lesion staging, demonstrating a prominent growth of a lesion in the upper middle part of the left kidney, extending beyond the kidney and infiltrating the pancreatic body. The lesion exhibited significantly high FDG uptake (SUVmax = 10.2, MTV = 136.13 cm^3^, TLG = 484.62) and had a maximum dimension of approximately 6.8 cm × 8.9 cm (Figure 2).

**Figure 3:**
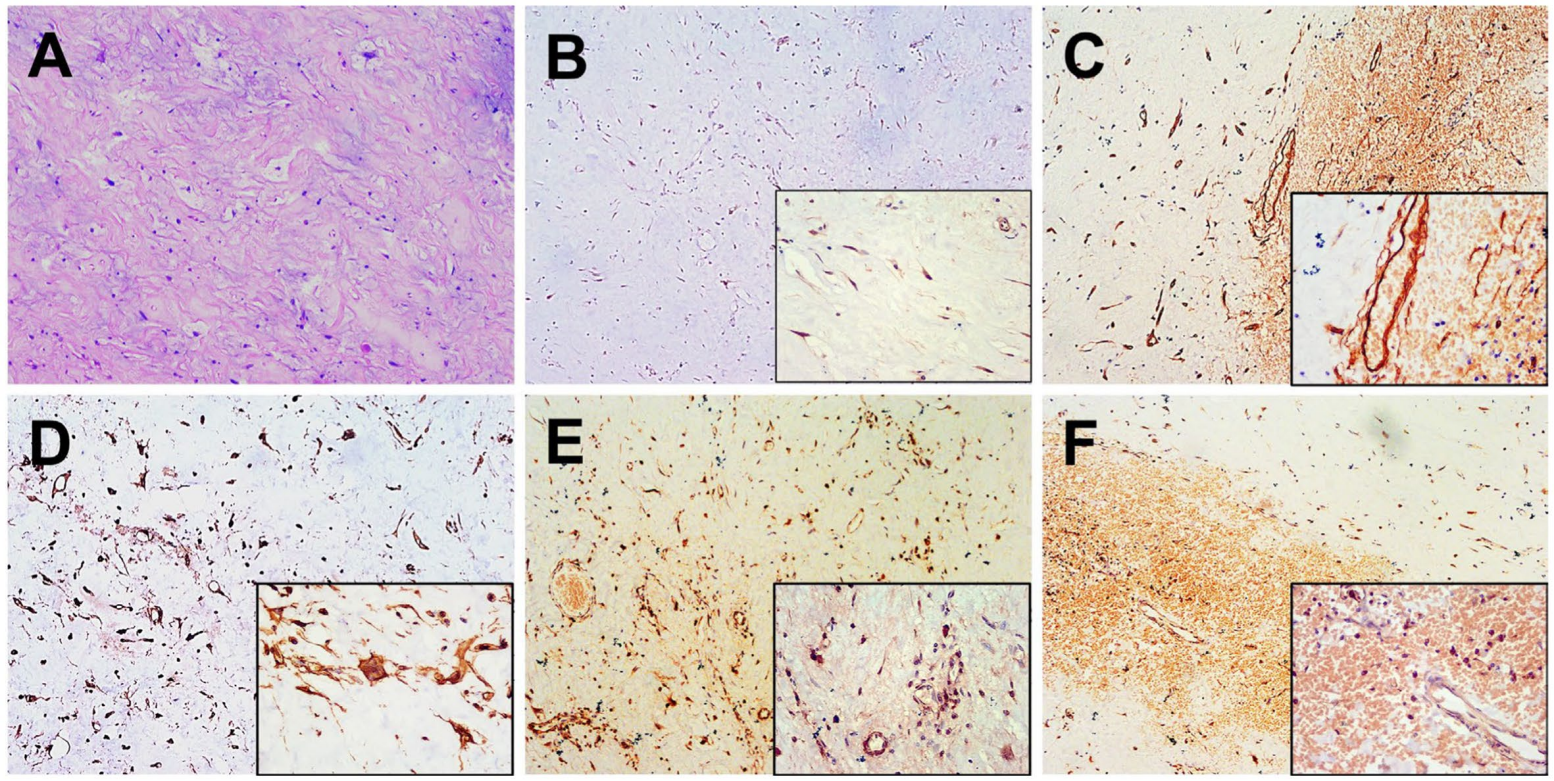
Histopathological and immunohistochemical images. **(A)** Hematoxylin–eosin (HE) staining (magnification ×100) showed a diffuse distribution of tumor cells and a mucus-like stroma, along with a significant presence of proliferating small vessels and abundant cytoplasm. Immunohistochemistry showed that the short spindle cells were positive for MDM2 **(B)**, CD34 **(C)**, Vimentin **(D)**, S-100 **(E)** and STAT-6 **(F)** was observed to be positive of the tumor cells (magnification ×40 and ×200).

The patient underwent a percutaneous pathology biopsy of the lesion, which resulted in a diagnosis of liposarcoma. Subsequently, the patient underwent surgical resection, revealing a left renal mass with dimensions of approximately 8.0 cm × 7.0 cm × 5.0 cm. The mass exhibited a grayish-yellowish-grayish-red color and a soft texture. Additionally, an exophytic growth of the tumor was observed, invading the pancreatic body, with a pancreatic lesion measuring approximately 6.5 cm × 5.5 cm × 3.0 cm. Hematoxylin and eosin staining of the lesion demonstrated a diffuse distribution of tumor cells, with round cell areas accounting for approximately 10% of the tumor cells. Notably, frequent nuclear atypia and a mucus-like stroma were observed, along with a significant presence of proliferating small vessels and abundant cytoplasm (Figure 3A). Immunohistochemical results exhibited that the MDM2, CD34, Vimentin, S-100, and STAT-6 were positive in tumor cells (Figures 3B–F). The postoperative pathological examination confirmed the diagnosis of myxoid liposarcoma (MLS).

**Figure 4:**
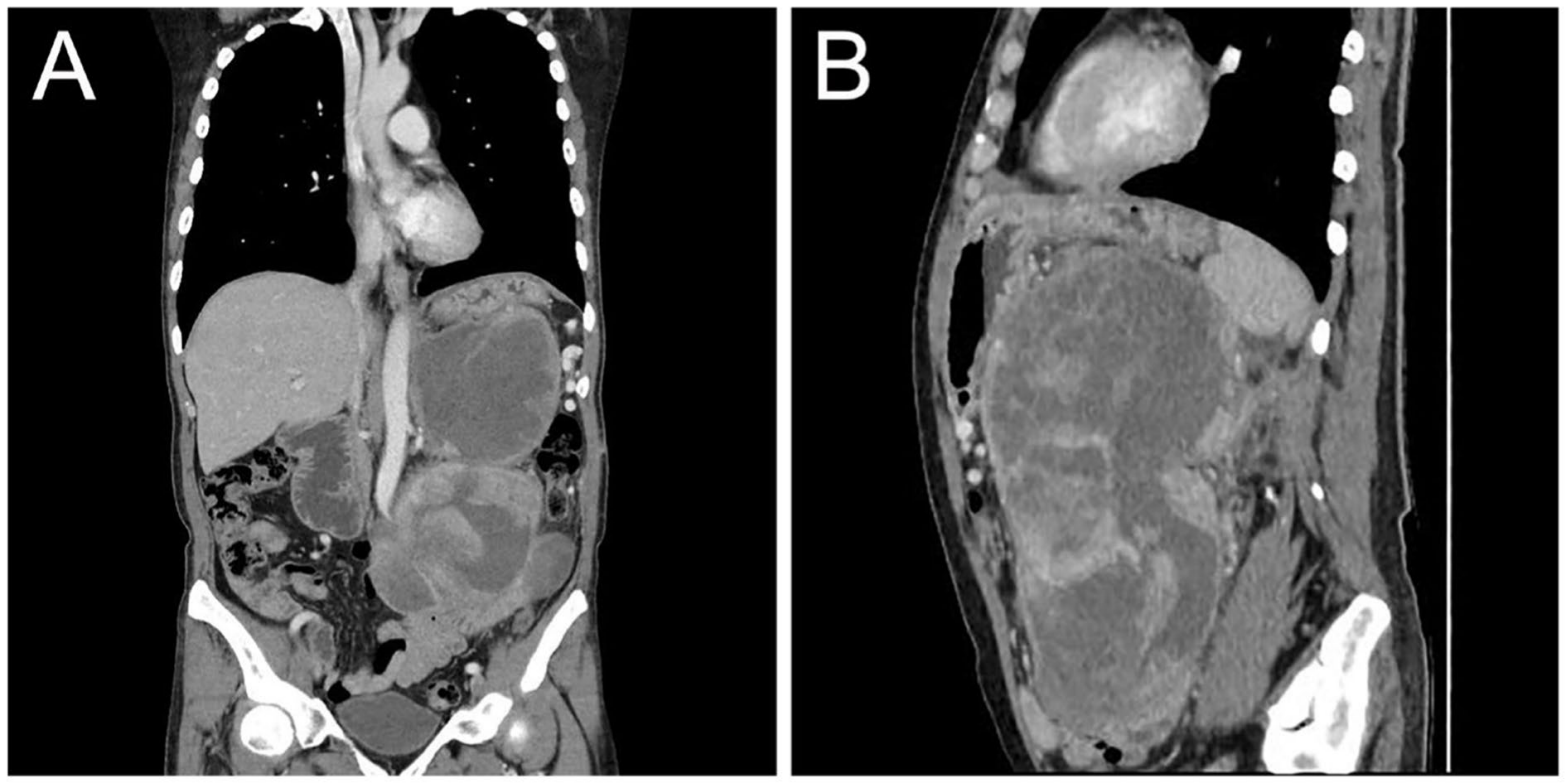
Postoperative contrast-enhanced CT images showed recurrence of MLS. Coronal **(A)** and sagittal **(B)** images obtained from the venous phase showed moderate inhomogeneous enhancement of the recurrent tumor in the abdominal cavity with compression and displacement of adjacent organs.

On the 10th day after surgery, the patient presented with symptoms of abdominal distension and vomiting. Physical examination revealed a palpable solid mass in the left upper abdomen, measuring approximately 20 cm × 15 cm, with limited mobility and tenderness upon pressure. Subsequent CT examination confirmed tumor recurrence (Figure 4). Despite the lack of the intended chemotherapy regimen, the patient underwent another surgical resection, revealing a retroperitoneal mass measuring approximately 20 cm × 15 cm. The mass invaded the pancreas, transverse colonic mesentery, duodenum, and spleen. Unfortunately, at the conclusion of the operation, the patient experienced a sudden cardiac arrest and could not be revived despite aggressive resuscitation efforts. The cardiac arrest was considered to be associated with the patient’s previous history of cardiac disease.

## Discussion

The kidney is an uncommon site for MLS. In our case, MLS originated from the fatty component of the left renal sinus, and its infiltrative growth invaded the pancreas, which is exceptionally rare. The patient presented with symptoms of abdominal distention and vomiting, attributed to the rapid tumor growth exerting pressure on the stomach.

Chromosomal translocations represent the most prevalent molecular abnormalities identified in sarcomas (30). MLS is distinguished by the *t(12:16) (q13:p11)* chromosomal translocation, which occurs in 95% of patients and gives rise to the FUS-CHOP oncoprotein (10). In a minority of cases, the *EWSR1* gene on chromosome 22 may be implicated, resulting in the *EWSR1-DDIT3* fusion. The diagnosis of MLS relies on histopathological features, which include a nodular growth pattern characterized by a combination of uniform non-lipogenic cells and small lipoblasts exhibiting a signet-ring appearance. These cells are found within a prominent myxoid stroma that is characterized by an abundance of hyaluronic acid and distinctive plexiform vasculature (31). Occasionally, metaplastic cartilaginous and osseous elements may be observed. High-grade MLS is defined as tumors in which the round cell component constitutes over 5% of the total tumor (3). While immunohistochemistry is not essential for the differential diagnosis, molecular investigations should be performed to explore specific mutations. In this case, both MDM2 and CD34 were positive, and the definitive diagnosis was primarily based on HE-stained histology.

The diagnosis of MLS poses an enduring challenge (26). Imaging modalities, including ultrasound, CT, MRI, and PET scans, serve as invaluable tools for tumor identification (32). However, there is currently no established standard regarding the timing and type of imaging surveillance in MLS (9). MLS is distinguished by its distinct pattern of metastatic spread, underscoring the importance of detecting metastatic disease to determine prognosis and guide management decisions (5). In our case, the CT features of MLS included an extra-renal distending, infiltrative growth invading the pancreas with cystic necrosis. Multiple renal artery branches were observed entering the mass, consistent with the pathology of a mucus-like interstitial stroma rich in vascular structures, thereby demonstrating noticeable enhancement. While the presence of fat content is a characteristic feature of MLS, histologically, the mucus component predominates and the fat content is minimal. Consequently, the fat component was not distinctly evident on CT in this particular case. Several studies have documented the substantial clinical efficacy of ^18^F-FDG PET/CT in the initial diagnosis, staging, biopsy site selection, restaging, and prediction of prognosis and response to therapy in the management of soft tissue sarcomas (33–37). We have summarized the previously reported manifestations of MLS using ^18^F-FDG PET/CT in Table 1 (19–29), which revealed low ^18^F-FDG activity ranging from (0.79 to 5.5, median, 2.535). The utility of FDG PET/CT in tumor follow-up has been questioned in some reports (16, 18). However, our case demonstrates a notable ^18^F-FDG uptake associated with a high-grade pathology (with at least a 10% round cell component), showing increased ^18^F-FDG uptake by round cells. A unique advantage of ^18^F-FDG PET/CT was its ability to confirm that the tumor in our case originated from the renal sinus, with direct invasion of the pancreas, thereby excluding the diagnosis of metastatic MLS.

Renal MLS should be differentiated from clear cell renal cell carcinoma (RCC) and renal leiomyosarcoma (RLMS). Clear cell RCC represents approximately 75%–80% of all cases of renal cell carcinoma and originates from the epithelium of the proximal tubule. Typically, clear cell RCC manifests as a heterogeneous mass in the renal cortex, characterized by rapid enhancement followed by contrast wash-out (38, 39). On MRI, T1WI demonstrates iso-or hypo-signal intensity, while T2WI shows inhomogeneous high signal (40, 41). Additionally, there is high ^18^F-FDG uptake on PET/CT (42). RLMS is an aggressive mesenchymal tumor that typically originates from the smooth muscle cells of the intrarenal blood vessels or the renal pelvis (43). CT scans reveal isointensity or slight hyperintensity, along with delayed or persistent enhancement. Tumors frequently exhibit external extension, easily encircling and infiltrating the inferior vena cava, while also showing a tendency to form tumor emboli and develop distant lymph node metastasis. MRI can provide additional insight into the tumor composition, elucidating hemorrhage and necrosis at different stages. On T1WI, the tumor shows iso-signal intensity, while on T2WI, there is distinct hypointensity (44). Furthermore, ^18^F-FDG PET/CT demonstrates increased uptake (45). Therefore, from an imaging perspective, our case is not easily distinguishable from clear cell RCC and RLMS, necessitating a reliance on pathological diagnosis.

The utility of ^18^F-FDG PET in evaluating liposarcomas has been well-established. According to a meta-analysis conducted by Ioannidis and Lau (46), which included 15 studies comprising 441 soft-tissue tumors, PET/CT demonstrated positive results in all intermediate-and high-grade liposarcomas (95% confidence interval 97.3–100). Brenner et al. (47) discovered that SUVmax has the potential to be clinically useful for risk stratification and patient management in liposarcoma. In their study, the SUVmax for MLS was determined to be 3.5 ± 1.5, and a tumor SUVmax exceeding the group mean value of 3.6 was significantly associated with reduced disease-free survival and served as an indicator for identifying patients at a high risk of developing early local recurrences or metastases. ^18^F-FDG PET/CT plays a significant role in determining treatment and follow-up strategies in MLS by detecting early metastatic disease and highlighting primary and metastatic soft tissue sites (19, 48). Ozguven et al. (29) performed ^18^F-FDG PET/CT for restaging a patient with MLS, revealing multiple metastases in the mesenteric region. This finding led to a change in the therapeutic management, with the addition of systemic chemotherapy to the wide surgical excision of the gluteal mass. Yokouchi et al. (49) reported the detection of a solitary metastatic breast tumor arising from MLS of the lower limbs using ^18^F-FDG PET/CT. Liu et al. (24) utilized ^18^F-FDG PET/CT for a comprehensive evaluation of primary hepatic MLS, confirming its origin and ruling out a metastatic diagnosis. Additionally, ^18^F-FDG PET/CT may serve as a valuable non-invasive modality for malignant grading and differentiating between subtypes of liposarcoma. Suzuki et al. (21) demonstrated that the mean ^18^F-FDG SUVs of MLS and other types of liposarcoma were significantly higher than those of well-differentiated liposarcoma by two-and three-fold, respectively. These accumulation rates were remarkably well related to their biological malignant grades.

Several prognostic factors have been identified in MLS, including age over 45 years, tumor size exceeding 10 cm, round cell differentiation exceeding 5%, and the presence of tumor necrosis, all of which are associated with a poor prognosis (50). Surgical resection remains the primary treatment option for MLS (51–53), with the resection margin playing a crucial role in patient survival (54). Inadequate excision is considered the primary cause of local recurrence. Both radiotherapy and chemotherapy are employed in the management of MLS (55, 56). Radiotherapy is administered both pre-and post-operatively for early-stage disease and for palliative purposes in cases of metastatic disease. Chemotherapy is primarily utilized for palliation in cases of metastatic disease. A phase II clinical trial investigating neoadjuvant trabectedin in patients with advanced localized MLS demonstrated significant efficacy and minimal toxicity, using a dosage of 1.5 mg/m^2^ trabectedin every 3 weeks (57). Unfortunately, our patient experienced early recurrence, had not yet undergone chemotherapy, and succumbed to the disease following re-surgical resection.

## Conclusion

In conclusion, we present an exceptionally rare case of MLS involving the left kidney with invasion into the pancreas. Due to the limited research on the utilization of ^18^F-FDG PET/CT in this particular context, given the rarity and low incidence of MLS, its role remains largely unexplored. However, as PET/CT imaging becomes increasingly prevalent, a comprehensive assessment of disease sites becomes indispensable for the development of treatment protocols and the monitoring of treatment response. This capability also enables the exploration of various interventions in terms of their type and timing, with the potential to enhance patient outcomes and mitigate morbidity and mortality.

## Data availability statement

The original contributions presented in the study are included in the article/supplementary material, further inquiries can be directed to the corresponding author.

## Ethics statement

Written informed consent was obtained from the individual(s) for the publication of any potentially identifiable images or data included in this article.

## Author contributions

WH and FC: manuscript draft and editing. LL and YG: imaging data collection. YZ and YQ: imaging data analysis. JG: supervision. LK: writing-review and editing. All authors contributed to the article and approved the submitted version.

## Funding

This work was supported by the National Natural Science Foundation of China (82171970), the Beijing Science Foundation for Distinguished Young Scholars (JQ21025), the Beijing Municipal Science & Technology Commission (Z221100007422027), National High Level Hospital Clinical Research Funding (Interdisciplinary Research Project of Peking University First Hospital, 2023IR17).

## Conflict of interest

The authors declare that the research was conducted in the absence of any commercial or financial relationships that could be construed as a potential conflict of interest.

## Publisher’s note

All claims expressed in this article are solely those of the authors and do not necessarily represent those of their affiliated organizations, or those of the publisher, the editors and the reviewers. Any product that may be evaluated in this article, or claim that may be made by its manufacturer, is not guaranteed or endorsed by the publisher.
